# Role of Ionizing Radiation in Neurodegenerative Diseases

**DOI:** 10.3389/fnagi.2018.00134

**Published:** 2018-05-14

**Authors:** Neel K. Sharma, Rupali Sharma, Deepali Mathur, Shashwat Sharad, Gillipsie Minhas, Kulsajan Bhatia, Akshay Anand, Sanchita P. Ghosh

**Affiliations:** ^1^Armed Forces Radiobiology Research Institute, Uniformed Services University of the Health Sciences, Bethesda, MD, United States; ^2^Center for Neuroscience and Regenerative Medicine, Department of Pathology, Uniformed Services University of the Health Sciences, Bethesda, MD, United States; ^3^Neurobiology Laboratory, School of Biotechnology, KIIT University, Bhubaneswar, India; ^4^Center for Prostate Disease Research, Department of Surgery, Uniformed Services University of the Health Sciences, Bethesda, MD, United States; ^5^Neuroscience Research Lab, Department of Neurology, Postgraduate Institute of Medical Education & Research (PGIMER), Chandigarh, India; ^6^Government Medical College and Hospital, Chandigarh, India

**Keywords:** ionizing radiation, hypoxia, aging, neurodegenerative diseases, CNS

## Abstract

Ionizing radiation (IR) from terrestrial sources is continually an unprotected peril to human beings. However, the medical radiation and global radiation background are main contributors to human exposure and causes of radiation sickness. At high-dose exposures acute radiation sickness occurs, whereas chronic effects may persist for a number of years. Radiation can increase many circulatory, age related and neurodegenerative diseases. Neurodegenerative diseases occur a long time after exposure to radiation, as demonstrated in atomic bomb survivors, and are still controversial. This review discuss the role of IR in neurodegenerative diseases and proposes an association between neurodegenerative diseases and exposure to IR.

## Introduction

The ionizing radiation (IR) is a group of subatomic particles and electromagnetic waves, or photons, which bear the capability to create electrically charged particles such as alpha, beta, gamma rays, and X-rays. The biological impact of IR on humans have already been reported a decade ago but recently there has been growing interest to understand the effect of radiation exposure in the central nervous system (CNS) in the clinical setting (Valentin, 2005). In today’s world with global threat of radiation exposure from warfare, the neurobiological impact of high and low doses of IR and responses to biological systems needs to be revaluated. In the brain, the effect of IR is widely seen in the Hippocampus, a radio-sensitive region of the brain which hosts proliferating progenitor cells (Harada et al., 2014; Pospisil et al., 2015). It has been shown that differentiating cells amalgamated into the hippocampal network leads to apoptosis or dysfunction due to exposure to high doses of irradiation and lead to changes in synaptic protein levels, dendritic complexity, morphology and spine density alterations. (Parihar and Limoli, 2013). According to the data generated by radiation research, as well as the guidelines of regulatory bodies, the safe dose is considered to be acute exposure to less than 100 mSv, or 0.1 gray (Gy) (Morgan and Bair, 2013).

**FIGURE 1:**
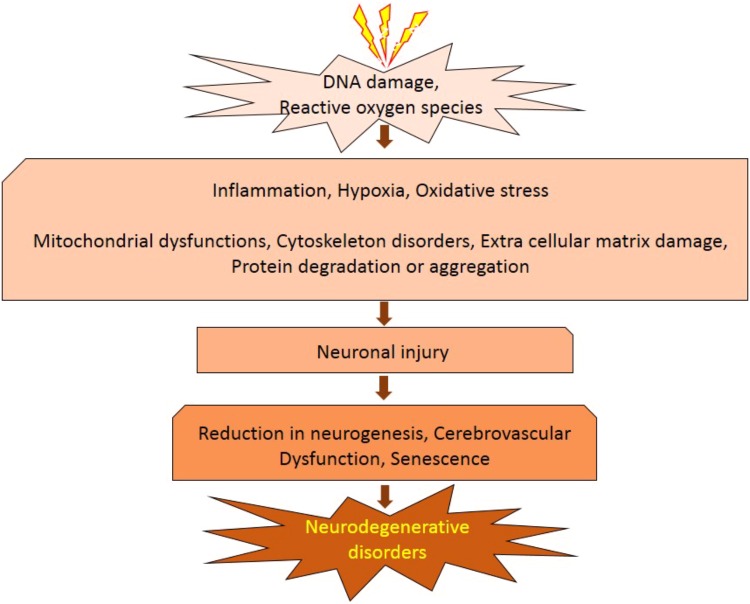
Ionizing radiation cause neural injury leads to neurodegenerative diseases.

**FIGURE 2:**
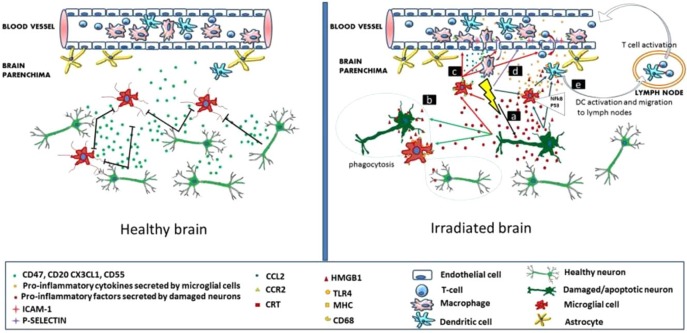
Inflammation-mediated mechanisms in radiation induced brain injury. **(Left)** Healthy brain secretes CD55, CD47, CX3CL1, CD20 and have intact neurons. **(Right)** Radiations affect the neurons and microglia in brain. Reproduced with permission (Lumniczky et al., 2017).

It has been established that radiation inhibits neurogenesis in a dose dependent manner (Low to High; >2 Gy to 45 Gy) through radiosensitive populations of neural stem and progenitor cells housing in the sub-granular zone of the dentate gyrus of the brain (Acharya et al., 2015). This can block the generation of new cells in the brain and cause neuroinflammation (Belarbi et al., 2013; Greene-Schloesser et al., 2013). Further, low dose exposures of IR have been shown to elevate the reactive oxygen and nitrogen species, which has the potential to initiate the changes in the redox balance of the CNS microenvironment (Limoli et al., 2004). Acute exposure of IR has manifold effects on brain and cognitive functions (Loganovsky, 2009), which can directly act and manipulate the nervous system and indirectly damage the other systems through CNS reactivity (Kimeldorf and Hunt, 1965; Mickley, 1987). Both low and high doses of radiation can alter the function of CNS by oxidative stress, mitochondrial dysfunctions and protein degradation, leading to senescence or apoptotic cell death which can cause defects leading to neurodegenerative diseases (**Figure 1**). In brain, inflammatory reactions are induced by IR via microglia and endothelial cell activation. Microglial cells can be activated (MHC, CD68 upregulation) due to IR-induced double-strand breaks, which activates the NFκB pathway-mediated creation of proteins related to inflammation (**Figure 2**) (Lumniczky et al., 2017). High-mobility group protein 1 (HMGB1) in the extracellular environment are secreted by damaged neurons, which is a ligand for TLR4 on the activated microglia. Calreticulin is expressed by damaged neurons on the surface which is detected by activated microglia and produce phagocytosis of both healthy and damaged neurons. Activated microglia also increases the secretion of chemokine (C-C motif) ligand 2 (CCL2) and its receptor. The peripheral macrophages expressing C-C chemokine receptor type 2 (CCR2) penetrate the blood–brain barrier and the adhesion markers like intercellular adhesion molecule 1 (ICAM-1), P-selectin starts upregulating on endothelial cells of brain after radiation. HMGB1 and pro-inflammatory signals are emitted by impaired neurons and activated microglial cells activate brain-residing dendritic cells, which migrate to regional lymph nodes and induce immune activation in the brain (**Figure 2**) (Lumniczky et al., 2017). Neurogenesis in the hippocampus is inhibited by pro-inflammatory cytokines which are secreted by activated Microglial cells and disturb neurogenic signaling pathways. Kim et al. (2008) reported that acute radiation sickness in adult ICR mice disrupts the hippocampus functioning, which includes learning and memory, through neurogenesis inhibition. Raber et al. (2004), suggested that a decrease in neurogenesis in the pathogenesis of IR-induced cognitive impairments is associated with a decrease in proliferating Ki-67-positive cells and double cortin-positive immature neurons in the subgranular zone (SGZ) of the dentate gyrus.

There are different stress response pathways by which cells respond to different stressful environments, such as unfolded protein response, heat shock response, DNA damage response, oxidative stress response, which help to maintain the cellular homeostasis, either by pro-survival pathways or by killing the damaged cells through apoptosis or autophagy (Fulda et al., 2010). Accumulation of misfolded proteins, such as those observed in neurodegenerative diseases like Alzheimer’s, Parkinson’s and Amyotrophic Lateral Sclerosis, is known to initiate stress response pathways to eliminate the aggregated proteins (Rao and Bredesen, 2004). It is also well established that the exposure to radiations causes misfolding and aggregation of proteins, which could further lead to activation of the unfolded protein response signaling pathway. There is evidence that radiation may speed up rates of folding and unfolding for globular proteins (de Pomerai et al., 2003). This can increase the chances of collision between partially unfolded molecules, leading to irreversible aggregation. The misfolding of protein leads to the protein aggregation which further develop the neurodegenerative disease. In our daily life, we are constantly exposed to the IR either from natural and/or man-made sources and it is known that IR exposure contributes to the etiology of neurodegenerative diseases (**Table 1**). In this review, we discussed the role of IR exposure with several of these neurodegenerative diseases including Multiple sclerosis, Age related macular degeneration, Amyotrophic lateral sclerosis, Ischemia related degeneration and Parkinson disease.

**Table 1:** Late effects of radiation.

Source/Species	Late effects	Reference
Radiotherapy/human	Multiple lesions in periventricular area, centrum semiovale and corpus callosum were detected after magnetic resonance imaging. Developed Multiple sclerosis.	Shaygannejad et al., 2013
Radiotherapy/human	Magnetic resonance imaging showed new hyperintense lesions. Radiation treatment triggered an exacerbation of multiple sclerosis	Murphy et al., 2003
X-radiation/human	Activation of quiescent MS with plaques confined to the radiation fields. Multiple sclerosis activated by x-radiation	McMeekin et al., 1969
X-radiation/Between 4000 and 6000 rad (40–60 Gy)/human	Four patients who received radiation in full tumoricidal doses had unexpectedly poor clinical outcome, suggesting that radiation is especially injurious to patients with demyelinating disease.	Peterson et al., 1993
Gamma-irradiation (0.5-Gy) once a week for 4 weeks/mice	Findings demonstrated suppression of pro-inflammatory cytokines, reduction of cytotoxic T cells and induction of regulatory T cells in mice.	Tsukimoto et al., 2008
50 Hz magnetic fields at two intensities [100 and 1000 microT (rms)] for 7 weeks/mice	No link between exposure and ALS development	Poulletier de Gannes et al., 2009
X-ray irradiation at a dose of 0.8–1.5 Gy/min (Total dose of 4–16 Gy)/mice	No association between SOD1 mutation and radio-sensitivity	Wate et al., 2005
Dose-rate 1–2 Gy/min/Cells from ALS patients (Total 0 to 8 Gy)	No significant differences in production of DNA double-strand breaks	Mithal et al., 1999
Continuous radiation/mice (1.4 mGy/h) for 45 days	Chronic low-dose radiation exposure is genotoxic in mice	Graupner et al., 2016
Conventional radiotherapy in treatment/human	Direct relationship between radiation exposure and cerebrovascular events	Little, 2016
Gamma and x rays at dose greater than 0.1 Gy/human	Increased risk of stroke in those exposed to radiation more than 0.1 Gy	Azizova et al., 2014
X rays-0 to 30 Gy/human	Increased adhesiveness of human aortic endothelial cells which is chemokine mediated	Khaled et al., 2012
X rays and gamma rays in interventional procedures/human	Increased incidence of stroke noticed among workers	Rajaraman et al., 2016
Head and Neck Radiotherapy/human	Increased incidence of cerebrovascular events	Plummer et al., 2011
Longitudinal cohort studies of Japanese atomic bomb survivors, ionizing radiation	Increased incidence noted in cardiovascular diseases including stroke, Rheumatic heart disease (RHD), ischaemic heart disease (IHD), cardiomyopathy Heart failure and cerebral hemorrhage	Takahashi et al., 2013
Single radiation dose of 14 Gy/ApoE^-/-^ mouse	Irradiation accelerates the development of macrophage-rich, inflammatory atherosclerotic lesions prone to intraplaque hemorrhage	Stewart et al., 2006
Mean dose 97 mv followed by max of 909 mv gamma radiations	Increased risk for death. cerebrovascular incidents was recorded as compared to other cardiovascular events	Kreuzer et al., 2013
Irradiation of CNS with doses 0, 5, 15, 25, and 35 Gy	Increased ICAM-1 expression. Suggests that increased leukocyte trafficking into the CNS may exacerbate the inflammation induced by radiation injury.	Olschowka et al., 1997
Cath lab radiation exposure	Decreased telomerase length and increased intima thickness of carotids	Andreassi et al., 2015


## Role of Ionizing Radiation in DNA Damage and Effect on Neurodegeneration

Effect of IR on the human DNA have been implicated due to varying degree of penetrance. Exposure to IR directly damages DNA by inducing DNA breaks particularly double stranded breaks (DSBs), which prevent the DNA replication in growing cells and cause arrest in S-phase of the development cycle. DSBs are very much detrimental, which, if left unrepaired, may have harsh aftereffects and the cell cannot survive. It also leads to the formation of reactive oxygen species (ROS) which causes oxidative stress in cells and it is indirectly linked with DNA damage. However, there is a repair system in cell which is triggered when DNA is damaged, and it stops the cell cycle at specific checkpoints to repair DNA damage and prevent progression of the cell cycle. After DNA damage, signaling molecules like ataxia telangiectasia mutated (ATM) and RAD3-related (ATR) kinases are activated which are the central controller of DNA damage response signaling pathway. These kinases work collectively and regulate downstream processes. Furthermore, phosphorylation of the histone variant histone-2AX (H2AX), is activated. Phosphorylation of H2AX plays an essential role in DNA damage response and is needed for the aggregating DNA repair proteins at the sites containing damaged DNA as well as for activation of checkpoints proteins which arrest the cell cycle progression. Generation of ROS causes cell cycle dysregulation, decrease in cell viability and damages proteins and lipids by oxidizing them and eventually leading to cell death. Koturbash et al. (2016) reported that low dose exposure to IR results in DNA damage as indicated by increased occurrence of DSBs and also behavioral changes.

It has also been reported that low dose radiation changes the expression of genes implicated in cell cycle control and DNA synthesis/repair (Yin et al., 2003). Brain cells are non-proliferative in nature and may be susceptible to the progressive accumulation of unrepaired DNA lesions exposure to radiation at low doses (<50 cGy) significantly induces neurocognitive deficits, such as learning and behavioral changes. CNS behavioral changes such as chronic fatigue and depression occur in patients who undergoes irradiation for cancer therapy. At lower radiation doses, neurocognitive effects were especially observed in children. Radiation exposure is related to a decline in academic achievement, intelligence and performance intelligence quotient (IQ). Increasing attention in recent years has been paid to the role of DNA damage and repair in neurological diseases. Many neurodegenerative diseases are related to defects in DNA single-strand break repair or double-strand break repair. In Parkinson’s disease (PD) and Alzheimer’s disease (AD) DNA repair defect could cause an abnormal accumulation of spontaneously occurring DNA damage in neurons *in vivo*, resulting in their premature death. There is reduction of DSB repair proteins like: DNA-PKcs and Mre11-Rad50-Nbs1 (MRN) as a result of high levels of DNA strand breaks and decreased base excision repair (BER) activity in AD patients (Jacobsen et al., 2004; Shackelford, 2006). In Amyotrophic lateral sclerosis (ALS) patients, elevated levels of oxidative lesions and single-strand breaks (SSBs) have been reported in the neurons (Bender et al., 2006; Kraytsberg et al., 2006). To say that DNA damage has an underlying effect in the pathology of neurodegenerative diseases, requires stipulating what lesions and if any have tendency to accumulate in the sick neurons, they need to be classify with the molecular mechanisms that prevent the repair of these lesions.

## Role of Ionizing Radiation in Multiple Sclerosis

Multiple sclerosis (MS) is a non-traumatic neurological disability commonly found among young people, with more than two million people affected worldwide (Sadovnick and Ebers, 1993). It’s a complex and an unpredictable disease of the CNS with an unknown etiology. Immune mediated demyelination, gliosis and axonal degeneration constitute major pathological features of MS (Mayr et al., 2003; Ascherio and Munger, 2008). MS is generally regarded as an autoimmune disease, since the body’s natural immunological defense destroys the body’s native cells and damages the myelin sheath (Fernandes de Abreu et al., 2009) instead of destroying foreign cells. In the United States alone, it is estimated that 85 per every 100,000 people suffer from MS (Noonan et al., 2002) and the disease occurs twice as frequently in women compared to men (Ascherio and Munger, 2008). Studies have shown that myelin damage occurs in both white matter and the cortical gray matter of MS patients (Geurts and Barkhof, 2008; Calabrese et al., 2010). Since the prevalence of MS appears to be increasing worldwide, it is imperative to elucidate the various contributing factors and molecular mechanisms to prevent the occurrence of MS. Environmental factors and genetic susceptibility are implicated in the pathogenesis of MS. However, IR is also contemplated as another potential factor of MS development (Flodin et al., 1988; Axelson et al., 2001; Bölviken, 2002). Although very few studies have been performed in the past to investigate the influence of IRs on the risk of MS development, existing studies suggests that IR may serve as a risk factor for MS.

Case control studies in Swedish population have demonstrated that individuals who are linked with radiological work and exhibit medical history of X-ray examinations have an increased risk for MS development (Bölviken, 2002). Tsukimoto et al. (2008) investigated the effect of low dose gamma irradiation (once a week for 4 weeks) on experimental allergic encephalomyelitis (EAE) animal model and observed a significant upregulation of regulatory T cells and suppression of proinflammatory cytokines such as inflammatory markers like interferon-γ (IFN-γ), interleukin 6 (IL-6), and interleukin 17 (IL-17). These findings imply that low dose gamma irradiation mitigates EAE through suppression of pro-inflammatory cytokines, reduction of cytotoxic T cells and induction of regulatory T cells.

A few reports have suggested that exposure to radiotherapy increases vascular permeability and may lead to the formation of demyelination lesions resulting in neuronal dysfunction (Lampert and Davis, 1964). In addition, chances of MS development may be elevated through exposure to cosmic rays, which are produced in northern and southern magnetic geographical areas (Barlow, 1960). Oldendorf and Cornford (1977) showed that spinal cord of rats exposed to X-radiation may aggravate symptoms of EAE (Oldendorf and Cornford, 1977).

Large numbers of individuals are exposed to natural sources of IR (e.g., cosmic radiation) as well as human-made radiation (e.g., X-rays and nuclear medicine) in day to day life. Accumulating data suggests that exposure to X-radiation may result in serious health concerns and adverse consequences if used intermittently and when doses of radiation exceed certain thresholds (Hammer et al., 2009; Bailey et al., 2010). Previous studies have investigated the potential hazards of IR and their effect on developing MS. In a case-control study, Motamed et al. (2014) determined the relationship between history of X-radiation and risk of MS. The investigators also determined whether the site and dosage of X-radiation would make any significant risk difference for MS (Motamed et al., 2014). Their findings revealed that patients who developed MS were previously exposed to X-ray radiation compared to controls, and that the difference was statistically significant. In addition, both cumulative number and dosage of X-radiation was significantly higher in MS patients compared to the controls. Their findings also demonstrated a link between X-ray radiation and risk of MS development in female patients, corroborating the notion that MS more frequently occurs in females. **Table 1** depicts source, dosage and effects of IR. A similar study from Sweden reported that MS patients prior to diagnosis had undergone X-ray examination several times during a 5-year period compared to controls (Flodin et al., 1988). In this study, only five MS patients were exposed to therapeutic X–ray as compared to controls. X-radiation was performed to diagnose individuals who were in the initial phases of the disease and also to identify the causes of the initial manifestations. This was considered as initial interpretation of these findings. Findings of Motamed et al. (2014) revealed that cumulative number of X-ray episodes was also significantly higher among the MS patients as compared to controls. Several lines of evidence have suggested interplay of immune system and oxidative stress underpinning the mechanism of MS pathogenesis (Lassmann et al., 2007). The cumulative dose and quality of radiation are crucial parameters to invoke immune and inflammatory reactions in brain (Amundson, 2008). Tsukimoto et al. (2008) investigated the effect of low dose gamma irradiation (once a week for 4 weeks) on an EAE animal model and observed a significant upregulation of regulatory T cells and suppression of proinflammatory cytokines such as IFN-gamma, IL6, and IL17 (Tsukimoto et al., 2008). These findings imply that low dose gamma irradiation mitigates EAE through suppression of pro-inflammatory cytokines, reduction of cytotoxic T cells and induction of regulatory T cells. Few other investigations have shown that TH1 cell activity is significantly enhanced and the levels of cytokines like IFN-γ, TNF-α, IL-2, are upregulated even with low dose radiation exposure. The level of other cytokines such as IL-10 is reduced which eventually results in free radical formation and oxidative damage of tissues (Liu, 2003). All of these perturbations in immune cell activity and cytokine levels disrupt blood brain barrier integrity, ultimately resulting in myelin and axonal damage which is observed in demyelinating MS disease pathology (Gilgun-Sherki et al., 2004). Furthermore, experimental and clinical data have reported that low dose radiation impedes tumor growth, diminishes metastasis, as well as alleviates the suppression of immunity due to tumor burden. Alike other immune related and neurodegenerative disorders (Taylor et al., 1975), it has been suggested that people suffering with MS have an increased sensitivity to IR (X-rays and gamma rays), which might elicit demyelination process in patients vulnerable to MS (Gipps and Kidson, 1981). Motamed et al. (2014) showed that patients diagnosed with MS were exposed to a considerable number of X-radiation and brain CT scanning in the past and that the cumulative dosage of IR is statistically related to the risk of MS. However, there are technical problems, which may be faced by the investigators in interpreting the results. For instance, there is a risk of misclassification bias. It is uncertain when exactly the pathological and molecular alterations appear in MS patients. Similarly, it is ambiguous that controls are devoid of any pathological alterations. These findings could result in reverse causation.

It has been observed that the chances of development of MS are more common in females upon exposure to any kind of X-ray radiation and imaging. It might be due to the smaller sample size of the male subgroup, which leads to a lower statistical significance. This difference in sex distribution is normal where MS is twice more common in females than males. However, MS-related genes present on the X chromosome have not been found by genome-wide association studies (GWAS). This suggests that preponderance of MS development in females is due to their female-specific physiology and hormones secreted in females (Orton et al., 2006). Higher prevalence of MS in females suggests that sex is a factor which makes females more susceptible to IRs. A recent study by Shaygannejad et al. (2013) has reported a case of MS development after a patient was exposed to radiotherapy for the diagnosed meningioma. These results might be due to the influence of radiation on the blood brain barrier and the interaction between immune system antigens and white matter, and the formation of demyelination lesions. Their findings suggest that prevailing doses of radiation might trigger autoimmunity. In a similar case report, MS symptoms were elevated in a patient after radiotherapy for parotid carcinoma (Murphy et al., 2003). Moreover, a patient initially diagnosed for a glomus jugulare tumor showed an exacerbation of quiescent MS following radiotherapy (McMeekin et al., 1969). These results can be due to the reported development of disseminated plaques of demyelination, which is seen after radiotherapy (McMeekin et al., 1969). However, similar lesions can be seen in MS patients because of underlying a pre-disposition to demyelination.

The available data describing the effects of IR on the development of MS is meagre. Furthermore, the molecular mechanisms involved in radiation-induced MS are poorly understood. Some of the experiments conducted in animals and human case reports have suggested individuals who are considerably exposed to radiation are at a higher risk to develop MS symptoms. Even the impact of low dose of IR can be fatal and can cause serious health related problems if used intermittently. These findings indicate that IR may serve as confounding factor in MS disease. Therefore, it is of the utmost importance to better comprehend the possible relationship between exposure to IR and development of MS pathogenesis.

## Role of Ionizing Radiation in Alzheimer’s Disease

Five million people are estimated to live with Alzheimer’s disease (AD) in the United States, and it is estimated that by 2025 there will be 50% increase in AD patients (Hebert et al., 2004). In aging population AD is a primary cause of dementia (Ashford, 2004). Patients with AD experience symptoms including memory loss, cognitive alterations and behavioral changes (Budson and Price, 2005).

As AD global prevalence is predicted to be double after 20 years, so it is crucial to recognize the molecular pathogenesis and different contributing factors for AD prevention strategy. There are numerous evidence describing the effects of IR on the brain, suggesting that IR exposure may eventually favor the progress of AD.

After cleavage, amyloid precursor protein (APP) produces 4.5-kDa peptides known as amyloid-β (Aβ) protein which is related to the pathogenesis of AD (Sisodia and Price, 1995). Due to imbalance between the levels of Aβ production and clearance, abnormal accumulation of Aβ occurs and is associated with oxidative stress, neurofibrillary tangle (NFT) formation, neuronal loss (Calissano et al., 2009), inflammation (Wyss-Coray and Rogers, 2012), and ultimately results in AD-related cognitive impairment. Since neurons are resistant to radiation-induced cell killing, brain is considered to be comparatively resistant to IR. Though, there are several studies describing the role of various cognitive and physiological effects of IR at various doses. Lower doses can lead to cognitive dysfunction without making significant morphological modification, however, at higher doses microscopic changes are visible (Abayomi, 1996).

Cherry et al. (2012) examined the effects of Fe particle irradiation in mouse model of AD (APP/PS1). Author had shown that after 6 months exposure with Fe radiation at 1 GeV/μ (10 and 100 cGy) APP/PS1 mice showed reduced cognitive abilities measured by novel object recognition tests and contextual fear conditioning. Increase of Aβ plaque pathology in male mice was observed and ICAM-1 immunohistology showed endothelial activation after 100 cGy in male mice which was suggesting potential modifications in Aβ trafficking through the blood brain barrier as a possible cause of plaque increase. The outcomes from these experiments showed that the high charged particle radiation may rise Aβ plaque pathology in APP/PS1 mouse model of Alzheimer’s disease (Cherry et al., 2012).

Belka et al. (2001) suggested that IR leads to increased expression of IFN-γ and tumor necrosis factor-α (TNF-α), or adhesion molecules like ICAM-1 and E-selectin (Quarmby et al., 1999). Lowe et al. (2009) demonstrated that low dose of IR trigger gene modulation which is different than high dose and are associated with brain specific functions such as memory, learning and cognition. In agreement with the idea that IR low dose is a potential risk factor for AD, Lowe et al. (2009) have shown that the global gene variations in the irradiated mice brain were comparable to those detected in AD patients. Overall, changes by early IR involved ion regulation, signal transduction mechanisms and synaptic signaling; late changes involved metabolic functions, including myelin and protein synthesis.

Brain atrophy with neurologic and mental weakening has been recognized a few months later in radiation therapy patients without recurrent or residual brain tumors. Few reports suggest that dementia can be detected in 0% of long-term brain tumor survivors treated with radiotherapy (Imperato et al., 1990). Global damage of the cerebral white matter and progressive brain atrophy has been seen in magnetic resonance imaging (MRI) and computed tomography (CT) data (Asai et al., 1989). The neurological deficits of high-dose radiation are thought to be due to neural loss and demyelination with associated cognitive and neural deficiencies. Some of these cognitive defects after exposure to IR have been observed as a consequence of impaired neurogenesis.

Radiation effects in the CNS are more noticeable in kids than in adults; IR-induced cognitive effects comprise learning incapacities and are more pronounced in younger children. Studies from prenatally exposed atomic bombing of Hiroshima and Nagasaki particularly if that exposure occurs at critical stages in the growth of the neocortex stated that IR during gestation has harmful effects on the development of human brain.

Data on a variety of measures of cognitive function, including the occurrence of severe mental retardation as well as variation in the IQ and school performance, show significant effects on those survivors exposed 8–15 weeks and 16–25 weeks after ovulation. MRI from these mentally retarded survivors has revealed a large abnormally situated gray matter which suggest an abnormal migration of neurons lead to cognitive function of the brain (Schull and Otake, 1999). Rola et al. (2004) have irradiated (2–10 Gy) 21-day-old C57BL/J6 male mice brain to determine the acute radiosensitivity of the dentate subgranular zone and performed immunohistochemistry of the tissues harvested 48 h after radiation. Histopathological analysis of the tissues showed dose dependent decrease in immature neurons. For analyzing long term effects of radiation in the brain, mice were given a single dose of 5 Gy whole brain irradiation. Radiations significantly decreased the production of new neurons after one and 3 months, however, glial cells showed no change. Three months later irradiation, changes were observed in spatial memory retention deficits which provide evidence that young animal irradiation induces a long-term impairment of subgranular zone neurogenesis that is associated with hippocampal-dependent memory deficits (Rola et al., 2004). Effects on adult neurogenesis within the hippocampus may be related to such deficits. To investigate this, Ben et al have irradiated adult mice brain with 4 Gy single dose and observed 80% decrease after 48 h in the cells immunoreactive for the proliferation marker 16, Ki67. The number of pyknotic cells increased approximately 2.5 fold after 16 h. However, all these levels came to normal after few days. The radiation effect was reversible on proliferation and neurogenesis in the dentate gyrus (Ben Abdallah et al., 2007). It was shown that irradiation of 10-day-old mice at 8 Gy resulted in decreased hippocampal neurogenesis and afterward increased the susceptibility of the adult brain to hypoxia-ischemia (Zhu et al., 2009). IR to the immature brain produced long-lasting changes, including resulting in larger infarcts, decreased hippocampal neurogenesis, increased hemispheric tissue loss and more inflammation than in non-irradiated brains. Other IR effects on the brain include severe interruption of the blood–brain barrier (BBB) which is resulting from apoptosis induced by radiation of microvascular endothelial cells, as detected in rats and mice exposed to 50 Gy dose (Li et al., 2003). The molecular and cellular events that subtend these defects are still unknown although some development toward understanding has been occurred. Currently, there is no strong human patient data linking low IR exposure to increased AD. Little is known concerning the molecular mechanisms involved in radiation-induced dementia. Therefore, understanding the biological effects of IR at high and low doses is developing as major concern for neurological health. Further, research is now needed to understand more about the association between IR and the risk of developing Alzheimer’s.

## Role of Ionizing Radiation in Age Related Macular Degeneration

Age related macular degeneration (AMD) is common cause of blindness in all over the world (Sharma et al., 2013a). It is a multifactorial disease with major risk factors like hypertension, environmental, smoking and aging (Sharma et al., 2012, 2013b,c). Problems in choroidal circulation and endothelial cells play important role in the pathogenesis of AMD. IR play an important role in the damage of endothelial cells. Radiation causes oxidative stress which leads to ROS, vascular abnormalities and cause choroid circulation reduction (Peiretti et al., 2006). ROS induce serious damage to biomolecules. ROS attack structural and enzymatic proteins by the oxidation of prosthetic groups, residual amino acids, protein aggregates and formation of cross links as well as proteolysis. In the vital metabolic pathways the inactivation of key proteins can have serious consequences and can evoke single and double stranded DNA breaks which can lead to cell death and can expedite the process of age related macular degeneration. In the radiation model for cataract, post-translational modifications resulted in altered protein–protein interactions, and the formation of high-molecular-weight aggregates that were enriched for αB-crystallin.

**FIGURE 3:**
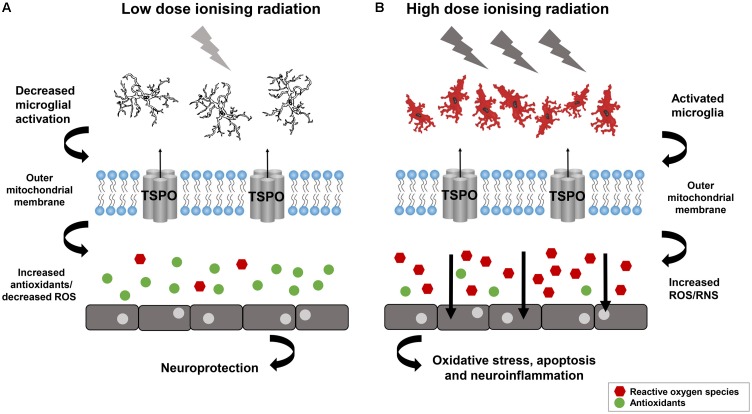
Low dose Ionizing radiation confer not only the neuroprotection but also activate reparative mechanisms. **(A)** Neuroinflammation may decrease by low dose ionizing radiation and can lead in increased antioxidants and reduced oxidative stress. **(B)** Neuroinflammation can be provoked by high dose ionizing radiation lead to microglia activation and ROS which can affect the cell functioning. Reproduced with permission (Betlazar et al., 2016).

There are significantly less studies on the role of IRs in causing AMD and effects in later life. Potential increase in morbidity and stroke after IRs also raises worry about risk of AMD. There is one report on Hiroshima and Nagasaki atomic bomb survivors in later life in which Itakura et al. (2015) investigated the relationship between atomic bomb exposure and the frequency of AMD. In 2006 to 2008, Itakura et al. (2015) selected 1824 participants to assess the prevalence of AMD in atomic bomb survivors. The eye dose for individuals was analyzed with DS02 (a revised dosimetry system) which took account for shielding conditions and physical locations at the time of explosion. For eyes, the absorbed dose was used in gray (Gy), for an individual the dose corresponds to the total exposure in gamma-rays +10 X the smaller neutron dose (Cullings et al., 2006). The estimated exposure dose for 43.6% individuals was ≤0.005 Gy and 4.8% were exposed to more than 2 Gy. Though they did not get any significant association with radiation dose and AMD prevalence, which suggested that long term oxidative stress after radiation may lead to suppression of neovascularization in the retina. The prevalence of neovascular AMD in their study was lower as compared to general population in Japan (Itakura et al., 2015). However, high doses of IR has been known to be susceptible for retinal vasculature for some time. The role of IR in protection and cause of diseases can be understand from **Figure 3** (Betlazar et al., 2016). Radiation retinopathy is slowly progressive microangiopathy and was first described by Stallard (1933). The lethal effects of IRs on retina was recognized in Moore (1935). Moore (1935) described the ischemic retinal vasculopathy in retinoblastoma patients treated by radium seeds.

Neuronal cells, particularly photoreceptors, are resistant to irradiation as compared to vascular endothelial cells. A dose dependent loss of pericytes in capillary regions and endothelial cells has been shown by histology. In later stages of life, vision loss is caused by retinal ischemia and retinal edema by secondary conditions, like retinal detachment, neurovascular glaucoma and vitreous hemorrhage. Additional obliterations can be seen in choriocapillaries in addition to inner retina. Archer et al demonstrated that radiation causes an exaggerated vasculopathy in diabetes mellitus patients, and diabetic rats (streptozotocin induced) develop ischaemic retinopathy after exposing to 1500 centigray (cGy) of radiation (Archer et al., 1991). Patients who receive a radiation dose of fewer than 2500 cGy in different fractions of 200 cGy are dubious to progress substantial retinopathy (Archer et al., 1991). Mild retinopathy patients progress gradually and visual functions remain almost normal, except if there is any substantial problem in macula. After irradiation at 10 or 15 Gy, wild type mice do not show any pathological change in the retina at any point in time, demonstrating that the wild type eye in mouse is not sensitive to single low dose of IR. However, after exposure to 18.5 Gy photoreceptors become obvious and with increase of dose at 22 and 25 Gy from an X-ray source, photoreceptors start terminating with damage to the retinal pigment epithelium containing large vacuoles (Gorgels et al., 2007). On the other hand, gamma rays at 10 Gy caused extensive retinal cell death in a Cockayne syndrome mouse model, a DNA repair disorder which suggests that oxidative DNA damage is responsible for the loss of photoreceptors (Gorgels et al., 2007). Amoaku et al. (1989) have demonstrated that the rat photoreceptor cells nuclei which are highly heterochromatic are intricately sensitive to IR as compared to those of the primate photoreceptors which are more euchromatic. Likewise, the inner retinal neurons – mainly ganglion-cells – are more euchromatic and revealed more resistant to radiation. It demonstrates that heterochromatin which is strongly packed is significantly more susceptible to damage after irradiation as compared to finely discrete euchromatin. This finding may clarify the effects of radiation on vascular endothelial-cells, whose nuclei are comparatively heterochromatic.

## Role of Ionizing Radiation in Amyotrophic Lateral Sclerosis

Amyotrophic lateral sclerosis (ALS) is a fatal neurodegenerative disease that has also been linked to radiation exposure. ALS is a motor-neuron disorder, where the neurons controlling the movement die. Various investigations have associated the risk of ALS with the exposure to radiation, primarily initiated with the high number of veterans reported with incidence of ALS related to exposure during service (Haley, 2003; Horner et al., 2003).

Majority of ALS cases reported are sporadic in nature, with just 10–20% cases connected with family history. In recent times, the studies of ALS and associated genetic mutations have gained pace, which has resulted in many discoveries. Mutations in different genes have been associated in past with the ALS, mainly in the gene encoding the antioxidant enzyme, superoxide dismutase 1 (SOD1), indicating important role of oxidative stress in ALS pathogenesis (Turner et al., 2013). Another gene that has been investigated with respect to ALS is the apurinic endonuclease or apurinic/apyrimidinic endodeoxyribonuclease 1 (APEX1), which has shown to be a part of DNA repair and plays a neuroprotective role against oxidative stress and exposure to IR (Vasko et al., 2011). The APEX1 mutants did not show any redox activity as evident from a Scottish population study, where sporadic ALS patients reported an amino acid change mutation in the APEX1 gene (Hayward et al., 1999). Another gene discovery that has associated IR exposure to ALS is the FUS gene. The knock-out of the FUS gene in different studies has revealed aberrant DNA repair machinery and hence, sensitivity toward radiation exposure (Hicks et al., 2000; Kuroda et al., 2000). It has been postulated that the FUS gene interacts with IR responsive gene, such as cyclin D1 (*CCND1*) and blocks protein translation. Therefore, any mutation in the FUS gene will prevent it from binding with the genes such as *CCND1* and cause protein accumulation as well as further toxicity in motor neurons and cell death (Wang et al., 2008). Consequently, the major abnormality being reported in the ALS becomes the abnormal DNA repair machinery and accumulation of the damaged DNA (Bradley and Krasin, 1982).

Many epidemiological studies also report the disease hazard to the employees in the radiation related industries. The correlation between electrical exposure and occurrence of ALS was first reported in Haynal and Regli (1964). In a study pertaining to IR, it has been shown that the gamma-irradiation exposure perceived during occupation, diagnosis or accident has genotoxic effects in irradiated mice (Graupner et al., 2016). On the contrary, there are a few studies which nullify any involvement of radiation exposure with the disease emergence (Parlett et al., 2011).

Some familial cases (fALS) are associated with mutations in SOD1 which is an antioxidant enzyme whose action is conserved in many mutant forms, it was reported that in motoneurons wtSOD1 was present in cytoplasm and nuclei, while mutant SOD1 was mainly cytoplasmic (Sau et al., 2007). Any disruption in the SOD activity responsible for misfolded protein clearance in the two subcellular compartments. Cells with G93A-SOD1 mutation exhibited more DNA damage compared with those expressing wtSOD1 (Sau et al., 2007). Mutant SOD1 toxicity might therefore arise from misfolding or destabilization of the protein. However, there are no point mutations in SOD1 linked to fALS, suggesting that the whole SOD1 protein is involved (Khare et al., 2005). Protein misfolding may then activate a cascade of actions which include protein buildup, possibly followed mitochondrial or proteasome dysfunctions and axonal transport alterations. Additionally, these events may indirectly lead to activation of caspase and ROSs (Boillee et al., 2006). All these events might be interconnected and thus worsening the severity of the preliminary trigger, the protein misfolding. Poulletier de Gannes et al. (2009) investigated the role of chronic exposure to electromagnetic radiation on the SOD1 mutant mouse model, the model used to mimic ALS. The study did not reveal any difference between the extremely-low frequency (ELF) magnetic fields exposure and development of ALS. Similarly, cells derived from the SOD1 mutant ALS mouse model were subjected to X-ray irradiation and the radio-sensitivity was assessed, which indicated no association between the SOD1 mutation and radio-sensitivity (Wate et al., 2005). In an identical study, cells isolated from patients with SOD1 mutations, when compared to sporadic ALS patients, revealed no difference in the production of DNA double-strand breaks after irradiation (Mithal et al., 1999). Further investigations are required to reveal a concrete correlation between the radiation exposure and ALS.

## Role of Ionizing Radiation in Ischemia Related Degeneration

Stroke is cerebrovascular disorder characterized by diminished blood supply to the brain. As a result, ischemic damage occurs, which is characterized by death of the brain cells resulting in plethora of signs and symptoms in concurrence with area of brain involved.

About 95 percent of stroke cases are present above the age of 65 years. Death rate and morbidity associated with stroke has increased with age and chances of recovery are minimal following a paralytic event. Since the incidence and prevalence of stroke has been increasing worldwide, it is important to highlight the emergent risk factors; especially targeting the role of IR. A case control showed direct causal relationship between the amount of dose and the cerebrovascular events in groups, which were treated by radiotherapy for non-malignant and malignant diseases and also in those exposed to environmental factors of radiation (Little, 2016). In another cohort study by Azizova et al. (2014) the incidence and mortality due to cerebrovascular diseases was assessed in 22,377 workers working in Mayak Production Association in 1948–1982 and were monitored up to 2008. These workers were subjected to occupational exposure to gamma and alpha rays. The mean plutonium body levels were calculated for both the types of radiation in males and females. Categorical analyses showed increased cerebrovascular disease incidence between employees with total absorbed external gamma-ray doses more than 0.1 Gy compared to lower doses exposed. The study shows direct linear relationship between risk of stroke and concentration of radiation exposure (Azizova et al., 2014). The immunological mechanism behind the pathogenesis of stroke is splendidly explained by Khaled et al. (2012). They exposed human aortic endothelial cells to 0–30 Gy x-rays and measured adhesiveness of endothelial cells using flow chamber assay. After 24 h of radiation, adhesiveness was increased with peak effect at 15 Gy. The study established a direct link between adhesiveness of chemokine associated endothelial cells which lead to subsequent increased risk of stroke (Khaled et al., 2012). In another study, Rajaraman et al. (2016) assessed the incidence and mortality among US medical radiation workers using fluorescent-guided interventional radiation procedures. A prospective cohort study was done with 90,957 technologists who were made to complete a survey regarding incidence of stroke among them spanning from 1994 to 2005. Thirty four percent increase in stroke was observed in technologists who were involved in fluoroscopically guided interventional procedures (Rajaraman et al., 2016). Apart from studies showing the environmental and occupational exposure of radiation various other studies indicated that routine treatment protocols have an equivocal say in the risk of stroke. As evident in study conducted by Plummer et al. (2011) demonstrated the effect of large dose radiotherapy to head and neck following head injury and trauma. Prospective and retrospective trials over past 30 years were conducted involving pathogenesis, imaging, epidemiology, and management of medium- and large-artery extra and intra cranial disease after neck and head radiotherapy. They concluded that neck and head radiotherapy rises the risks of stroke and transient ischemic attacks in the survivors (Plummer et al., 2011). MicroRNA’s (miRNA) are the non-specific RNAs, which cause post translational gene modification and increase the risk of certain malignancies. In a study conducted by Borghini et al. (2013), effect of low dose IRs in interventional cardiology were studied. These reports have demonstrated that using miRNA array, radiations cause dysregulation of brain specific miRNA and might prove to be having a role in pathogenesis of ischemic neurodegenration of brain tissue (Borghini et al., 2013). A study on German WISMUT uranium miners demonstrated the relationship among external gamma radiation and cerebrovascular diseases. The cohort study includes 58,982 former workers of the Wismut Company. During the follow up from 1946 to 2008 there were 9,039 deaths from cardiovascular diseases. Exposure to external gamma radiation was studied using job exposure matrix. The exposure was calculated using expert details till 1954 followed by measurements thereafter. Mean dose was 97 mv followed by max of 909 mv. Increased risk for death due to cerebrovascular incidents was recorded as compared to other cardiovascular events (Kreuzer et al., 2013).

Stewart et al. (2006) explained the effect of IR in developing atherosclerotic lesions in APOE mice and its risk of developing hemorrhage. They used a mouse model to study radiation induced atherosclerosis and compared it to age related plaque. Atherosclerosis prone APOE mice were subjected to radiation at the central vessels. At 22, 24, and 28 weeks, blood samples were taken and studied for changes in different markers. Cholesterol levels and inflammatory markers were not that much different from age-matched controls, however, there was increase in macrophages and marked influx of granulocytes predicting the role of inflammation. Intra-plaque hemorrhages and macrophage rich RBCs presence is prone to stroke like modality (Stewart et al., 2006). Injury to CNS results in inflammatory response, which is characterized by increased leukocyte activation and induction of cytokines. Injury model like stroke showed a marked influx of inflammatory mediators to CNS. This has been ascribed to increased levels of ICAM-1. In a mouse model, Olschowka et al. (1997) studied the relative induction of ICAM-1 using quantitative RT-PCR after 6 h following irradiation with either 0, 5, 15, 25, or 35 Gy and immunohistochemistry was done at 4, 24, 48 h and 7 days following 25 Gy irradiation. Results showed that the levels of ICAM1 and protein concentration increased (Olschowka et al., 1997) and suggested that activated ICAM-1 increases gene expression for proteins and resulted in increased risk for ICAM associated clotting which may increase risk of ischemic and hemorrhagic stroke.

After taking into consideration the human and natural sources of radiation, it is imminent to lay focus on the radiation exposure due to nuclear weapons detonation. In Japan, Takahashi et al. (2013) studied the impact of radiation on long-term survivors of atomic bomb at Hiroshima and the incidence of various cerebrovascular diseases in them. They documented that depending upon the age of exposure, density of exposure and the time of exposure to radiation from the atomic bombing, there was several fold increase in risk of certain cerebrovascular events like stroke, hemorrhages and myocardial infarction (MI) (Takahashi et al., 2013). A mathematical model constructed by researchers in the imperial college London predicts the role of low dose IR in cardiovascular events. They suggested that IR results in the killing of monocytes of the endothelial wall which would otherwise bind to MCP-1 whose levels increase in their absence. High levels of MCP-1 cause inflammation in the vessel wall resulting in damage to the cardiac tissue and may increase the risk of stroke via thrombus generation (Little et al., 2009). Stroke is a multifactorial disease, targeted by thrombotic, embolic and ischemic events. The risk of stroke and cerebrovascular insult increase with exposure to IR and the duration of exposure. Statistical studies discussed above have focused another aspect of stroke pathology related to radiation exposure. However, due to high background level of cerebrovascular accidents in civilian population, more emphasis should be laid on observing the overall impact of radiation by studying the animal models and co-relate them with human studies.

## Role of Ionizing Radiation in Parkinson’s Disease

Parkinson’s disease (PD) is the most common neurodegenerative disorder and its underlying molecular mechanisms are not fully understood. Aging in combination with the environmental and genetic factors plays a key role in the etiology of PD (Jenner et al., 2013; Xu and Chan, 2015). Environmental factors, such as pesticides, herbicide, metal irons and IR are the significant risk factors for PD (Xu and Chan, 2015). Inflammation and oxidative stress have also been associated with PD (Wang et al., 2013). The IR induces the inflammation and stress toward the neurobiological responses (Betlazar et al., 2016) and can cause PD. The most common defect of CNS is the neurogenic process in the brain and CNS is very sensitive to chemotherapy and IR (Acharya et al., 2015). IR exposure can lead to radiolytic lesions through various cellular process increasing the global stress response and impacting the DNA repair, cell cycle progression, and survival (Fike et al., 2007, 2009; Tseng et al., 2014). The IR response to neural precursor cells in the hippocampal dentate gyrus cell, altered neurogenesis suggesting to play a critical role in cognitive impairment (Mizumatsu et al., 2003). Further, the learning and memory deficiencies caused by the hippocampal dysfunction resulting into a long-term absence of normal stem/progenitor activity by IR (Monje and Palmer, 2003).

In our routine life, we are constantly exposed to the IR either from natural and/or man-made sources and it is known that IR exposure contributes to the etiology of neurodegenerative diseases (Kempf et al., 2013). PD is a progressive neurodegenerative disease and oxidative stress is involved in the progression of PD. The increase in ROSs damages the target neuronal cells (Hirsch, 1993) and dopaminergic neurons are more susceptible to oxidative stress. Further, the mitochondria plays an important role to maintain the cellular energy and calcium homeostasis. Any mitochondrial dysfunction results to energy and calcium imbalances, leads to cell death (Wang and Michaelis, 2010). It has been established that dopamine metabolism and mitochondrial dysfunctions are the leading cause elevated ROS in PD progression (Greenamyre and Hastings, 2004; Licker et al., 2009).

The known etiology of PD is to bear the alterations at molecular level when and compared to effect of low dose IR such as less than 5 Gy (Kempf et al., 2013). The children and young adults are more prone to IR and the role of X-rays and CT scans cannot be ignored as one of contributor of IR (Kempf et al., 2013). Thus, it is very important to analyze the *in vitro* and/or *in vivo* radiation data regardless of radiation source and age of animal models or human subjects.

It has been known that low doses of IR leads to mitochondrial dysfunction either by interacting with mitochondrial DNA (mtDNA) or through the formation of reactive hydroxyl radicals (Prithivirajsingh et al., 2004). Both the IR-induced damage and mtDNA alterations are important and critical to induce mitochondrial impairment to cause neurodegeneration. Therefore, mitochondria is the marked target for the late onset damage of low dose radiation. It has also been shown that after low-dose gamma-ray irradiation (0.5 Gy) in C57BL/6 mice brain, the antioxidant molecules, glutathione (GSH) and thioredoxin remained, elevated upto 12 h (Prithivirajsingh et al., 2004). This also increases the oxidative stress in PD due to depletion of total GSH followed by mitochondrial dearth (Jenner, 1993). The radiation-induced damage to mtDNA affects the mitochondrial synthesis (Malakhova et al., 2005). Further, the protein thiol moieties are considered as a key target of radiation-induced oxidation via ROS pathway (Winterbourn and Hampton, 2008). The imbalance in mitochondrial activity leads to neurodegeneration in PD (Arduino et al., 2011) and this destruction develops at early stage of PD (Bueler, 2009). Recently, Kobashigawa et al. (2011) showed that dynamin-related protein-1 (Drp1), a major regulatory component to manage the mitochondrial fission rate accumulated in mitochondria of normal human fibroblast cell after exposure to 6 Gy gamma radiations (Kobashigawa et al., 2011). Furthermore, after IR exposure, the loss of mitochondrial membrane leads to neuronal cell death in PD due to impaired oxidative phosphorylation and increase in apoptosis (Kobashigawa et al., 2011).

In the neuropathology animal model system, low dose IR confer not only the neuroprotection but also activate reparative mechanisms (**Figure 3**) (Betlazar et al., 2016). The PD model, “1-methyl-4-phenyl-1,2,3,6-tetrahydropyridine (MPTP)” showed increase in the levels of glutathione and catalase after 3 h exposure to 0.5 Gy gamma radiation (Yamaoka et al., 2002), which is further confirmed using mouse model of PD after whole body gamma radiation at 1.5 Gy (El-Ghazaly et al., 2015). The human epidemiological and animal model data suggest that the neurotoxin, MPTP and the pesticide, rotenone can trigger Parkinson like symptoms by inhibiting the mitochondrial Complex I and activating the ROS production demonstrating that the dysfunction of the mitochondria is a characteristic of PD (Coskun et al., 2012). The role of DNA damage in PD has also been shown in a cell culture based model system developed by Robbins et al. (1985). The lymphoblastoid cell line developed from the patient with sporadic PD were irradiated and they showed the genetic defect of DNA damage caused by somatic mutation during embryogenesis. This DNA repair defect leads to the abnormal accumulation of DNA damage in PD (Robbins et al., 1985). The radiosensitivity of sporadic PD patients’ cell lines which does not involve the patients’ germ cells showed premature death of their neurons *in vivo* due to genetic defect arising from dominant somatic mutation cropping up during embryogenesis (Robbins et al., 1985). The DNA damage caused by normally occurring DNA-damaging cellular metabolites, ROS, and spontaneous hydrolytic reactions provide the platform for the *in vitro* radiosensitivity and *in vivo* premature death of neurons in PD (Bankers Life and Casualty Company, National Cancer Institute (US), and International Symposium on Aging and Cancer, 1982). *In vitro* X-rays exposure of cells results in several unrepaired lethal lesions in DNA leads to PD. The *in vitro* radiosensitivity study of cultured non-neural cells from PD disease patients showed lethal abnormality elucidating the underlying mechanisms responsible for PD. The different clinical and neuropathological patterns of PD resulted from different defective repair processes originated from different mutations or DNA damage (Robbins et al., 1985). These findings indicate that mitochondria is a direct target of IR and mitochondrial defects leads to the development of PD.

Radiations may speed up the folding and unfolding of the proteins. It has long been known that the stability of proteins with respect to denaturation (as defined by aggregation) is lowered upon treatment with IR. α-synuclein, the main element of Lewy bodies, is highly conserved presynaptic protein linked to both familial and sporadic PD. The molecular mechanism for PD is strongly associated with α-synuclein aggregation (Breydo et al., 2012). The abnormal aggregation of α-synuclein in neurons leads to development of PD (Gundersen, 2010). Previously it was shown that α-synuclein protein is highly susceptible to dityrosine (DiY) crosslinking in protein by UV irradiation, resulting in DiY-modified α- synuclein monomers and dimers (Wordehoff et al., 2017). Radiation-induced oxidative stress can cause compromised mitochondrial functioning, protein misfolding and endoplasmic reticulum (ER) stress, besides DNA damage. Due to stress, parkin gene can also misfold the protein similar to α-synuclein. Martin et al. (1993) observed an increase of striatal D1 and D2 dopamin receptor density in discrete cerebral areas of rats, 2 h after exposure to (neutron-gamma) radiation at the dose of 5.5 or 7.5 Gy (Martin et al., 1993). The radiation may cause the α-synuclein aggregation, parkin gene misfolding and dopamine metabolism which may lead to inappropriate mitochondrial activity, Lewy bodies and cellular stress, the key component for the PD (Robinson, 2008).

## Conclusion

More detailed epidemiological studies as well as a better understanding of biological mechanisms are needed according to the evidences presented here in this review, which may address the misclassifying factors. The risks of circulatory, age related and neurodegenerative diseases are similar to those of radiation-induced cancers as reported for non-cancer diseases. Health effects after deep space radiation continue for long periods after exposure. Many circulatory diseases were observed in atomic bomb survivors in Japan at low doses as 0.5–2.0 Gy. Developing age related diseases after exposure to therapeutic and diagnostic purposes is always a health concern. However, slow growing tissues like brain require long exposure and high doses for developing degenerative symptoms. Different kinds of radiant energy cause diverse health effects ranging from birth defects to age related diseases decades after exposure, which depend up on the kind and conditions of radiation exposure. To understand the mechanism of radiation exposure in age-related and neurodegenerative diseases, further long term studies are needed.

## Author Contributions

NS and SG conceptualized, designed, edited the manuscript, and wrote the manuscript. RS edited the manuscript and contributed content for introduction and conclusion. DM contributed content for the Multiple sclerosis. GM contributed content for the ALS. SS contributed content for the PD. KB contributed content for the Stroke. AA edited and conceptualized the manuscript.

## Disclaimer

The opinions contained herein are the private views of the authors, and are not necessarily those of Armed Forces Radiobiology Research Institute, the Uniformed Services of the University of the Health Sciences, or the Department of Defense.

## Conflict of Interest Statement

The authors declare that the research was conducted in the absence of any commercial or financial relationships that could be construed as a potential conflict of interest.
